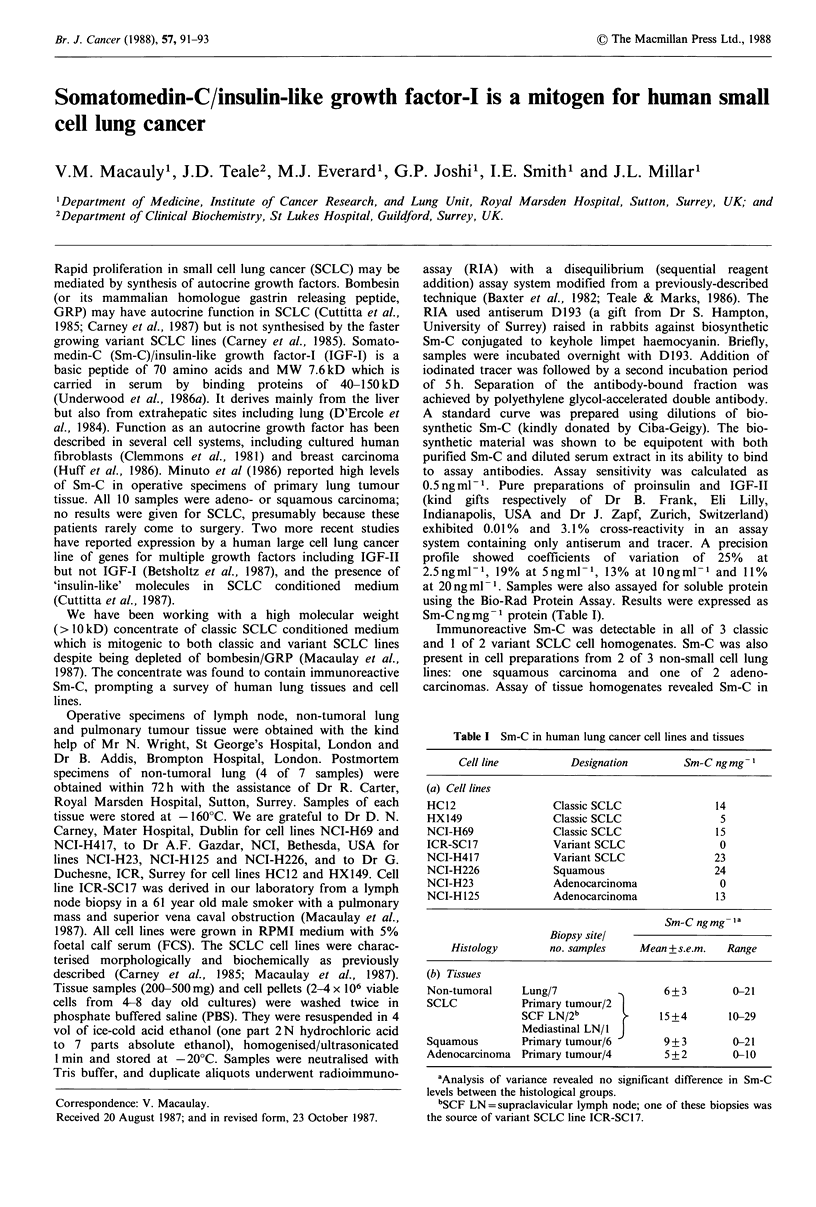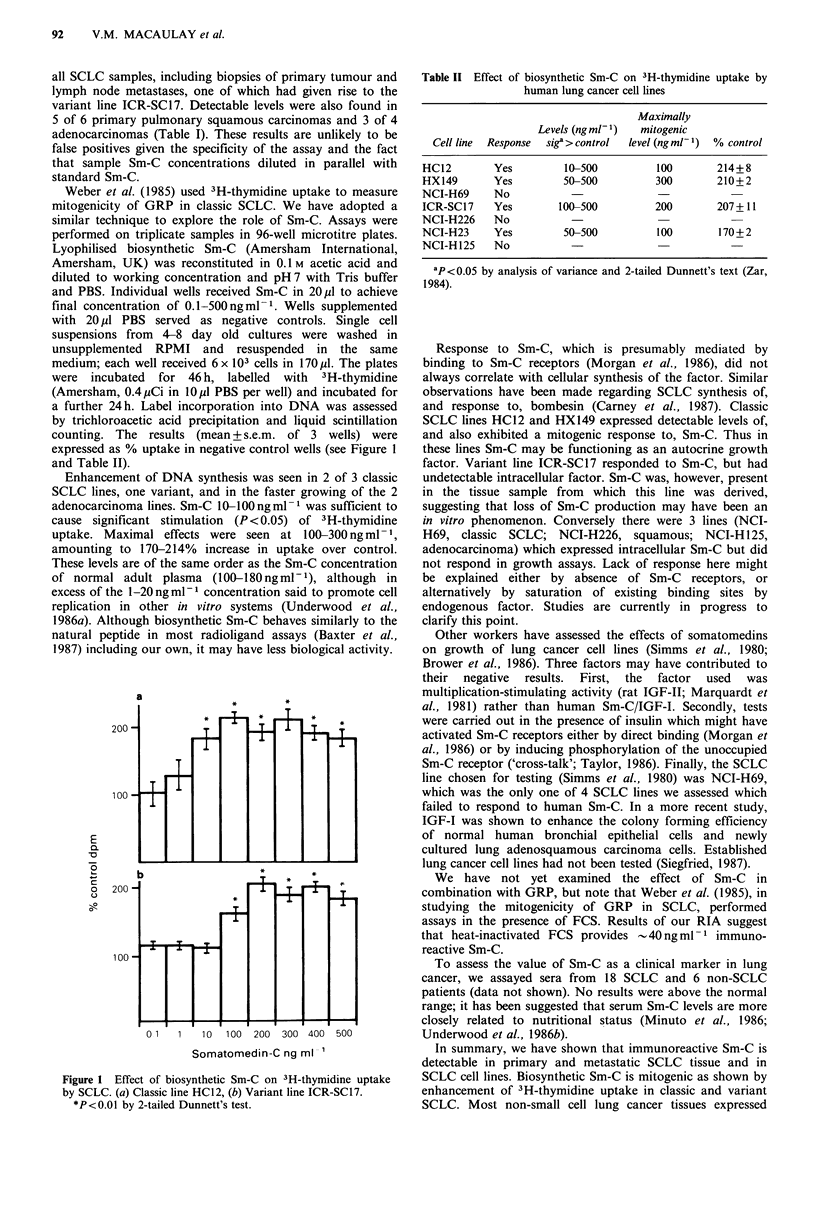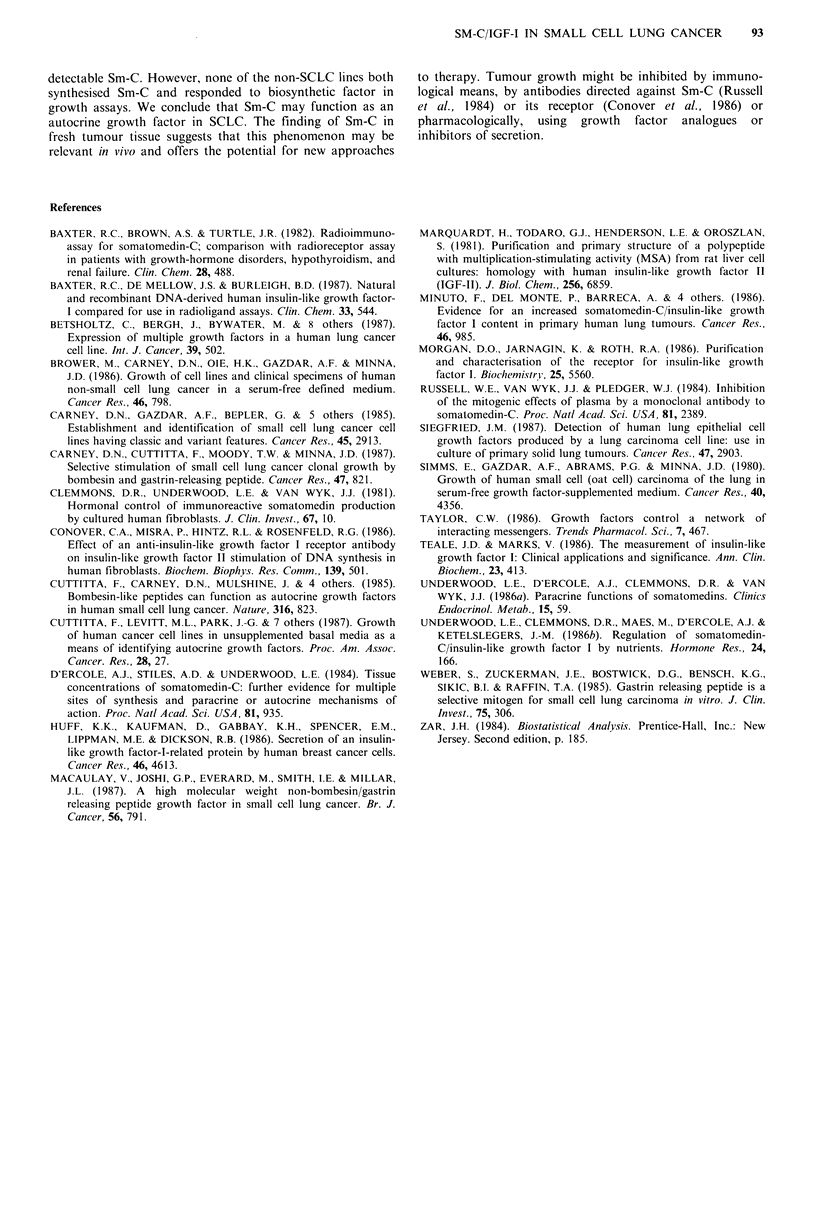# Somatomedin-C/insulin-like growth factor-I is a mitogen for human small cell lung cancer.

**DOI:** 10.1038/bjc.1988.16

**Published:** 1988-01

**Authors:** V. M. Macauly, J. D. Teale, M. J. Everard, G. P. Joshi, I. E. Smith, J. L. Millar

**Affiliations:** Department of Medicine, Royal Marsden Hospital, Sutton, Surrey, UK.


*Br. J. Cancer* (1988), **57**, 91–93 © The Macmillan Press Ltd., 1988

### Somatomedin-C/insulin-like growth factor-I is a mitogen for human small cell lung cancer

V.M. Macauly[1], J.D. Teale[2], M.J. Everard[1], G.P. Joshi[1], I.E. Smith[1] and J.L. Millar[1]

[1]*Department of Medicine, Institute of Cancer Research, and Lung Unit, Royal Marsden Hospital, Sutton, Surrey, UK; and* [2]*Department of Clinical Biochemistry, St Lukes Hospital, Guildford, Surrey, UK.*

Rapid proliferation in small cell lung cancer (SCLC) may be mediated by synthesis of autocrine growth factors. Bombesin (or its mammalian homologue gastrin releasing peptide, GRP) may have autocrine function in SCLC (Cuttitta *et al.*, 1985; Carney *et al.*, 1987) but is not synthesised by the faster growing variant SCLC lines (Carney *et al.*, 1985). Somatomedin-C (Sm-C)/insulin-like growth factor-I (IGF-I) is a basic peptide of 70 amino acids and MW 7.6kD which is carried in serum by binding proteins of 40–150kD (Underwood *et al.*, 1986*a*). It derives mainly from the liver but also from extrahepatic sites including lung (D'Ercole *et al.*, 1984). Function as an autocrine growth factor has been described in several cell systems, including cultured human fibroblasts (Clemmons *et al.*, 1981) and breast carcinoma (Huff *et al.*, 1986). Minuto *et al* (1986) reported high levels of Sm-C in operative specimens of primary lung tumour tissue. All 10 samples were adeno- or squamous carcinoma; no results were given for SCLC, presumably because these patients rarely come to surgery. Two more recent studies have reported expression by a human large cell lung cancer line of genes for multiple growth factors including IGF-II but not IGF-I (Betsholtz *et al.*, 1987), and the presence of 'insulin-like' molecules in SCLC conditioned medium (Cuttitta *et al.*, 1987).

We have been working with a high molecular weight (>10kD) concentrate of classic SCLC conditioned medium which is mitogenic to both classic and variant SCLC lines despite being depleted of bombesin/GRP (Macaulay *et al.*, 1987). The concentrate was found to contain immunoreactive Sm-C, prompting a survey of human lung tissues and cell lines.

Operative specimens of lymph node, non-tumoral lung and pulmonary tumour tissue were obtained with the kind help of Mr N. Wright, St George's Hospital, London and Dr B. Addis, Brompton Hospital, London. Postmortem specimens of non-tumoral lung (4 of 7 samples) were obtained within 72h with the assistance of Dr R. Carter, Royal Marsden Hospital, Sutton, Surrey. Samples of each tissue were stored at $-160$°C. We are grateful to Dr D. N. Carney, Mater Hospital, Dublin for cell lines NCI-H69 and NCI-H417, to Dr A.F. Gazdar, NCI, Bethesda, USA for lines NCI-H23, NCI-H125 and NCI-H226, and to Dr G. Duchesne, ICR, Surrey for cell lines HC12 and HX149. Cell line ICR-SC17 was derived in our laboratory from a lymph node biopsy in a 61 year old male smoker with a pulmonary mass and superior vena caval obstruction (Macaulay *et al.*, 1987). All cell lines were grown in RPMI medium with 5% foetal calf serum (FCS). The SCLC cell lines were characterised morphologically and biochemically as previously described (Carney *et al.*, 1985; Macaulay *et al.*, 1987). Tissue samples (200–500mg) and cell pellets ($2$–$4\times10^6$ viable cells from 4–8 day old cultures) were washed twice in phosphate buffered saline (PBS). They were resuspended in 4 vol of ice-cold acid ethanol (one part 2N hydrochloric acid to 7 parts absolute ethanol), homogenised/ultrasonicated 1min and stored at $-20$°C. Samples were neutralised with Tris buffer, and duplicate aliquots underwent radioimmunoassay (RIA) with a disequilibrium (sequential reagent addition) assay system modified from a previously-described technique (Baxter *et al.*, 1982; Teale & Marks, 1986). The RIA used antiserum D193 (a gift from Dr S. Hampton, University of Surrey) raised in rabbits against biosynthetic Sm-C conjugated to keyhole limpet haemocyanin. Briefly, samples were incubated overnight with D193. Addition of iodinated tracer was followed by a second incubation period of 5h. Separation of the antibody-bound fraction was achieved by polyethylene glycol-accelerated double antibody. A standard curve was prepared using dilutions of biosynthetic Sm-C (kindly donated by Ciba-Geigy). The biosynthetic material was shown to be equipotent with both purified Sm-C and diluted serum extract in its ability to bind to assay antibodies. Assay sensitivity was calculated as $0.5\,ng\,ml^{-1}$. Pure preparations of proinsulin and IGF-II (kind gifts respectively of Dr B. Frank, Eli Lilly, Indianapolis, USA and Dr J. Zapf, Zurich, Switzerland) exhibited 0.01% and 3.1% cross-reactivity in an assay system containing only antiserum and tracer. A precision profile showed coefficients of variation of 25% at $2.5\,ng\,ml^{-1}$, 19% at $5\,ng\,ml^{-1}$, 13% at $10\,ng\,ml^{-1}$ and 11% at $20\,ng\,ml^{-1}$. Samples were also assayed for soluble protein using the Bio-Rad Protein Assay. Results were expressed as Sm-C $ng\,mg^{-1}$ protein (Table I).

Immunoreactive Sm-C was detectable in all of 3 classic and 1 of 2 variant SCLC cell homogenates. Sm-C was also present in cell preparations from 2 of 3 non-small cell lung lines: one squamous carcinoma and one of 2 adenocarcinomas. Assay of tissue homogenates revealed Sm-C in

**Table I** Sm-C in human lung cancer cell lines and tissues

| *Cell line* | *Designation* | *Sm-C ng mg*$^{-1}$ |
|---|---|---|
| (*a*) *Cell lines* | | |
| HC12 | Classic SCLC | 14 |
| HX149 | Classic SCLC | 5 |
| NCI-H69 | Classic SCLC | 15 |
| ICR-SC17 | Variant SCLC | 0 |
| NCI-H417 | Variant SCLC | 23 |
| NCI-H226 | Squamous | 24 |
| NCI-H23 | Adenocarcinoma | 0 |
| NCI-H125 | Adenocarcinoma | 13 |

| *Histology* | *Biopsy site/ no. samples* | *Sm-C ng mg*$^{-1a}$ *Mean*±*s.e.m.* | *Range* |
|---|---|---|---|
| (*b*) *Tissues* | | | |
| Non-tumoral | Lung/7 | 6±3 | 0–21 |
| SCLC | Primary tumour/2; SCF LN/2[b]; Mediastinal LN/1 | 15±4 | 10–29 |
| Squamous | Primary tumour/6 | 9±3 | 0–21 |
| Adenocarcinoma | Primary tumour/4 | 5±2 | 0–10 |

[a]Analysis of variance revealed no significant difference in Sm-C levels between the histological groups.
[b]SCF LN = supraclavicular lymph node; one of these biopsies was the source of variant SCLC line ICR-SC17.

Correspondence: V. Macaulay.
Received 20 August 1987; and in revised form, 23 October 1987.

all SCLC samples, including biopsies of primary tumour and lymph node metastases, one of which had given rise to the variant line ICR-SC17. Detectable levels were also found in 5 of 6 primary pulmonary squamous carcinomas and 3 of 4 adenocarcinomas (Table I). These results are unlikely to be false positives given the specificity of the assay and the fact that sample Sm-C concentrations diluted in parallel with standard Sm-C.

Weber *et al.* (1985) used $^3$H-thymidine uptake to measure mitogenicity of GRP in classic SCLC. We have adopted a similar technique to explore the role of Sm-C. Assays were performed on triplicate samples in 96-well microtitre plates. Lyophilised biosynthetic Sm-C (Amersham International, Amersham, UK) was reconstituted in 0.1 M acetic acid and diluted to working concentration and pH 7 with Tris buffer and PBS. Individual wells received Sm-C in 20 $\mu$l to achieve final concentration of 0.1–500 ng ml$^{-1}$. Wells supplemented with 20 $\mu$l PBS served as negative controls. Single cell suspensions from 4–8 day old cultures were washed in unsupplemented RPMI and resuspended in the same medium; each well received $6 \times 10^3$ cells in 170 $\mu$l. The plates were incubated for 46 h, labelled with $^3$H-thymidine (Amersham, 0.4 $\mu$Ci in 10 $\mu$l PBS per well) and incubated for a further 24 h. Label incorporation into DNA was assessed by trichloroacetic acid precipitation and liquid scintillation counting. The results (mean $\pm$ s.e.m. of 3 wells) were expressed as % uptake in negative control wells (see Figure 1 and Table II).

Enhancement of DNA synthesis was seen in 2 of 3 classic SCLC lines, one variant, and in the faster growing of the 2 adenocarcinoma lines. Sm-C 10–100 ng ml$^{-1}$ was sufficient to cause significant stimulation ($P<0.05$) of $^3$H-thymidine uptake. Maximal effects were seen at 100–300 ng ml$^{-1}$, amounting to 170–214% increase in uptake over control. These levels are of the same order as the Sm-C concentration of normal adult plasma (100–180 ng ml$^{-1}$), although in excess of the 1–20 ng ml$^{-1}$ concentration said to promote cell replication in other *in vitro* systems (Underwood *et al.*, 1986*a*). Although biosynthetic Sm-C behaves similarly to the natural peptide in most radioligand assays (Baxter *et al.*, 1987) including our own, it may have less biological activity.

**Figure 1** Effect of biosynthetic Sm-C on $^3$H-thymidine uptake by SCLC. (*a*) Classic line HC12, (*b*) Variant line ICR-SC17. *$P<0.01$ by 2-tailed Dunnett's test.

**Table II** Effect of biosynthetic Sm-C on $^3$H-thymidine uptake by human lung cancer cell lines

| *Cell line* | *Response* | *Levels (ng ml$^{-1}$) sig$^a$ > control* | *Maximally mitogenic level (ng ml$^{-1}$)* | *% control* |
|---|---|---|---|---|
| HC12 | Yes | 10–500 | 100 | 214±8 |
| HX149 | Yes | 50–500 | 300 | 210±2 |
| NCI-H69 | No | — | — | — |
| ICR-SC17 | Yes | 100–500 | 200 | 207±11 |
| NCI-H226 | No | — | — | — |
| NCI-H23 | Yes | 50–500 | 100 | 170±2 |
| NCI-H125 | No | — | — | — |

[a] $P<0.05$ by analysis of variance and 2-tailed Dunnett's text (Zar, 1984).

Response to Sm-C, which is presumably mediated by binding to Sm-C receptors (Morgan *et al.*, 1986), did not always correlate with cellular synthesis of the factor. Similar observations have been made regarding SCLC synthesis of, and response to, bombesin (Carney *et al.*, 1987). Classic SCLC lines HC12 and HX149 expressed detectable levels of, and also exhibited a mitogenic response to, Sm-C. Thus in these lines Sm-C may be functioning as an autocrine growth factor. Variant line ICR-SC17 responded to Sm-C, but had undetectable intracellular factor. Sm-C was, however, present in the tissue sample from which this line was derived, suggesting that loss of Sm-C production may have been an *in vitro* phenomenon. Conversely there were 3 lines (NCI-H69, classic SCLC; NCI-H226, squamous; NCI-H125, adenocarcinoma) which expressed intracellular Sm-C but did not respond in growth assays. Lack of response here might be explained either by absence of Sm-C receptors, or alternatively by saturation of existing binding sites by endogenous factor. Studies are currently in progress to clarify this point.

Other workers have assessed the effects of somatomedins on growth of lung cancer cell lines (Simms *et al.*, 1980; Brower *et al.*, 1986). Three factors may have contributed to their negative results. First, the factor used was multiplication-stimulating activity (rat IGF-II; Marquardt *et al.*, 1981) rather than human Sm-C/IGF-I. Secondly, tests were carried out in the presence of insulin which might have activated Sm-C receptors either by direct binding (Morgan *et al.*, 1986) or by inducing phosphorylation of the unoccupied Sm-C receptor ('cross-talk'; Taylor, 1986). Finally, the SCLC line chosen for testing (Simms *et al.*, 1980) was NCI-H69, which was the only one of 4 SCLC lines we assessed which failed to respond to human Sm-C. In a more recent study, IGF-I was shown to enhance the colony forming efficiency of normal human bronchial epithelial cells and newly cultured lung adenosquamous carcinoma cells. Established lung cancer cell lines had not been tested (Siegfried, 1987).

We have not yet examined the effect of Sm-C in combination with GRP, but note that Weber *et al.* (1985), in studying the mitogenicity of GRP in SCLC, performed assays in the presence of FCS. Results of our RIA suggest that heat-inactivated FCS provides ~40 ng ml$^{-1}$ immunoreactive Sm-C.

To assess the value of Sm-C as a clinical marker in lung cancer, we assayed sera from 18 SCLC and 6 non-SCLC patients (data not shown). No results were above the normal range; it has been suggested that serum Sm-C levels are more closely related to nutritional status (Minuto *et al.*, 1986; Underwood *et al.*, 1986*b*).

In summary, we have shown that immunoreactive Sm-C is detectable in primary and metastatic SCLC tissue and in SCLC cell lines. Biosynthetic Sm-C is mitogenic as shown by enhancement of $^3$H-thymidine uptake in classic and variant SCLC. Most non-small cell lung cancer tissues expressed detectable Sm-C. However, none of the non-SCLC lines both synthesised Sm-C and responded to biosynthetic factor in growth assays. We conclude that Sm-C may function as an autocrine growth factor in SCLC. The finding of Sm-C in fresh tumour tissue suggests that this phenomenon may be relevant *in vivo* and offers the potential for new approaches to therapy. Tumour growth might be inhibited by immunological means, by antibodies directed against Sm-C (Russell *et al.*, 1984) or its receptor (Conover *et al.*, 1986) or pharmacologically, using growth factor analogues or inhibitors of secretion.

### References

BAXTER, R.C., BROWN, A.S. & TURTLE, J.R. (1982). Radioimmunoassay for somatomedin-C; comparison with radioreceptor assay in patients with growth-hormone disorders, hypothyroidism, and renal failure. *Clin. Chem.* **28**, 488.

BAXTER, R.C., DE MELLOW, J.S. & BURLEIGH, B.D. (1987). Natural and recombinant DNA-derived human insulin-like growth factor-I compared for use in radioligand assays. *Clin. Chem.* **33**, 544.

BETSHOLTZ, C., BERGH, J., BYWATER, M. & 8 others (1987). Expression of multiple growth factors in a human lung cancer cell line. *Int. J. Cancer*, **39**, 502.

BROWER, M., CARNEY, D.N., OIE, H.K., GAZDAR, A.F. & MINNA, J.D. (1986). Growth of cell lines and clinical specimens of human non-small cell lung cancer in a serum-free defined medium. *Cancer Res.*, **46**, 798.

CARNEY, D.N., GAZDAR, A.F., BEPLER, G. & 5 others (1985). Establishment and identification of small cell lung cancer cell lines having classic and variant features. *Cancer Res.*, **45**, 2913.

CARNEY, D.N., CUTTITTA, F., MOODY, T.W. & MINNA, J.D. (1987). Selective stimulation of small cell lung cancer clonal growth by bombesin and gastrin-releasing peptide. *Cancer Res.*, **47**, 821.

CLEMMONS, D.R., UNDERWOOD, L.E. & VAN WYK, J.J. (1981). Hormonal control of immunoreactive somatomedin production by cultured human fibroblasts. *J. Clin. Invest.*, **67**, 10.

CONOVER, C.A., MISRA, P., HINTZ, R.L. & ROSENFELD, R.G. (1986). Effect of an anti-insulin-like growth factor I receptor antibody on insulin-like growth factor II stimulation of DNA synthesis in human fibroblasts. *Biochem. Biophys. Res. Comm.*, **139**, 501.

CUTTITTA, F., CARNEY, D.N., MULSHINE, J. & 4 others. (1985). Bombesin-like peptides can function as autocrine growth factors in human small cell lung cancer. *Nature*, **316**, 823.

CUTTITTA, F., LEVITT, M.L., PARK, J.-G. & 7 others (1987). Growth of human cancer cell lines in unsupplemented basal media as a means of identifying autocrine growth factors. *Proc. Am. Assoc. Cancer. Res.*, **28**, 27.

D'ERCOLE, A.J., STILES, A.D. & UNDERWOOD, L.E. (1984). Tissue concentrations of somatomedin-C: further evidence for multiple sites of synthesis and paracrine or autocrine mechanisms of action. *Proc. Natl Acad. Sci. USA*, **81**, 935.

HUFF, K.K., KAUFMAN, D., GABBAY, K.H., SPENCER, E.M., LIPPMAN, M.E. & DICKSON, R.B. (1986). Secretion of an insulin-like growth factor-I-related protein by human breast cancer cells. *Cancer Res.*, **46**, 4613.

MACAULAY, V., JOSHI, G.P., EVERARD, M., SMITH, I.E. & MILLAR, J.L. (1987). A high molecular weight non-bombesin/gastrin releasing peptide growth factor in small cell lung cancer. *Br. J. Cancer*, **56**, 791.

MARQUARDT, H., TODARO, G.J., HENDERSON, L.E. & OROSZLAN, S. (1981). Purification and primary structure of a polypeptide with multiplication-stimulating activity (MSA) from rat liver cell cultures: homology with human insulin-like growth factor II (IGF-II). *J. Biol. Chem.*, **256**, 6859.

MINUTO, F., DEL MONTE, P., BARRECA, A. & 4 others. (1986). Evidence for an increased somatomedin-C/insulin-like growth factor I content in primary human lung tumours. *Cancer Res.*, **46**, 985.

MORGAN, D.O., JARNAGIN, K. & ROTH, R.A. (1986). Purification and characterisation of the receptor for insulin-like growth factor I. *Biochemistry*, **25**, 5560.

RUSSELL, W.E., VAN WYK, J.J. & PLEDGER, W.J. (1984). Inhibition of the mitogenic effects of plasma by a monoclonal antibody to somatomedin-C. *Proc. Natl Acad. Sci. USA*, **81**, 2389.

SIEGFRIED, J.M. (1987). Detection of human lung epithelial cell growth factors produced by a lung carcinoma cell line: use in culture of primary solid lung tumours. *Cancer Res.*, **47**, 2903.

SIMMS, E., GAZDAR, A.F., ABRAMS, P.G. & MINNA, J.D. (1980). Growth of human small cell (oat cell) carcinoma of the lung in serum-free growth factor-supplemented medium. *Cancer Res.*, **40**, 4356.

TAYLOR, C.W. (1986). Growth factors control a network of interacting messengers. *Trends Pharmacol. Sci.*, **7**, 467.

TEALE, J.D. & MARKS, V. (1986). The measurement of insulin-like growth factor I: Clinical applications and significance. *Ann. Clin. Biochem.*, **23**, 413.

UNDERWOOD, L.E., D'ERCOLE, A.J., CLEMMONS, D.R. & VAN WYK, J.J. (1986*a*). Paracrine functions of somatomedins. *Clinics Endocrinol. Metab.*, **15**, 59.

UNDERWOOD, L.E., CLEMMONS, D.R., MAES, M., D'ERCOLE, A.J. & KETELSLEGERS, J.-M. (1986*b*). Regulation of somatomedin-C/insulin-like growth factor I by nutrients. *Hormone Res.*, **24**, 166.

WEBER, S., ZUCKERMAN, J.E., BOSTWICK, D.G., BENSCH, K.G., SIKIC, B.I. & RAFFIN, T.A. (1985). Gastrin releasing peptide is a selective mitogen for small cell lung carcinoma *in vitro*. *J. Clin. Invest.*, **75**, 306.

ZAR, J.H. (1984). *Biostatistical Analysis*. Prentice-Hall, Inc.: New Jersey. Second edition, p. 185.